# Navigating Evidence on Ultra-Processed Foods and Cancer: A Narrative Review

**DOI:** 10.7759/cureus.96173

**Published:** 2025-11-05

**Authors:** Sindhu Vithayathil, Maryam Walizada, Gowtham Siddi, Srirachana Reddy Gumireddy, Mariette Anto, Israa Elkashif, Ann Kashmer Yu

**Affiliations:** 1 Medicine, American University of Antigua, Coolidge, ATG; 2 Internal Medicine, Mary Washington Hospital, Fredericksburg, USA; 3 Medicine and Surgery, NRI Academy of Medical Sciences, Guntur, IND; 4 Medicine and Surgery, Apollo Institute of Medical Sciences and Research, Hyderabad, IND; 5 Orthopedics, Kings Mill Hospital, Sutton-in-Ashfield, GBR; 6 Psychiatry, Royal College of Surgeons in Ireland, Dublin, IRL; 7 Internal Medicine, California Institute of Behavioral Neurosciences and Psychology, Fairfield, USA

**Keywords:** cancer, cancer risk, food classification, food processing, ultra-processed food

## Abstract

A healthy human body is built on the foundation of proper nutrition. Ultra-processed foods (UPFs) are made with ingredients that are uncommon in home kitchens, such as preservatives, flavorings, emulsifiers, and refined substances. Globally, the consumption of UPFs continues to rise, while the intake of nutrient-dense foods declines. At the same time, cancer incidence is also increasing, particularly among younger populations. Although the exact reasons for this trend remain unclear, numerous studies are underway to better understand the contributing factors. One area of growing interest is the potential link between UPF consumption and cancer risk. We reviewed observational studies, systematic reviews, and literature reviews published in PubMed, PMC, and ScienceDirect between 2010 and 2025 to evaluate evidence for the association between UPF and cancer risk. A total of 663 articles were initially identified across the databases using our defined keywords. After duplicate removal and successive screening of titles, abstracts, and study quality, 11 research papers were included in the final review. Growing evidence focuses on several possible ways UPFs may influence cancer risk, such as exposing the body to harmful additives, disrupting the balance of gut bacteria, and triggering chronic systemic inflammation. Still, a definite cause-and-effect relationship has not been established. Overall, observational studies consistently demonstrate an association between the consumption of UPFs and an increased risk of cancer, although causality remains uncertain.

## Introduction and background

With 18.1 million new cases reported globally in 2020, cancer remains a critical public health concern, and its incidence is projected to climb to nearly 28 million cases per year by 2040 [1,2]. In the United States alone, over two million new cancer diagnoses are expected in 2025, with mortality exceeding 600,000 deaths per year [3]. While advancements in diagnostics and treatment have improved outcomes for many types of cancer, prevention remains a critical and cost-effective strategy [4]. Alongside well-established genetic and environmental risk factors, there is growing recognition of the role that modifiable lifestyle behaviors, particularly dietary patterns, play in cancer development and progression [4,5]. Diet is a key factor that influences cancer risk, treatment response, and survivorship, highlighting the vital role of nutrition in cancer prevention strategies [6]. Dietary patterns and food production systems have undergone profound transformations in both developed and developing nations [6,7]. Traditional diets, once centered on locally sourced fresh produce, tubers, and animal-based foods prepared at home, have increasingly been replaced by packaged, processed, and ready-to-eat or ready-to-heat products [7,8]. This rapid shift, often referred to as the “nutrition transition,” has significant implications for human biological adaptation, chronic disease risk, and environmental sustainability [7,9].

The dietary transformation is characterized by the sharp rise in the intake of ultra-processed foods (UPFs), defined by the NOVA classification system as industrial formulations with little or no resemblance to natural foods, typically energy-dense, high in added sugars, trans fats, sodium, and synthetic additives, while being poor in dietary fiber, vitamins, and minerals [10,11]. The methods and ingredients used in creating UPFs make them extremely convenient (ready-to-eat, long-lasting), highly appealing (deliciously addictive) to consumers, and very profitable (cheap ingredients, extended shelf life) for manufacturers [10]. Of the various definitions proposed for UPFs, the NOVA classification is regarded as the most specific, coherent, comprehensive, and practical [10,11]. The NOVA classification system provides a consistent and reproducible framework that classifies foods according to the degree and purpose of their processing, rather than solely by their nutrient composition [11]. It categorizes foods into four groups according to their degree of processing: unprocessed or minimally processed foods (e.g., fruits and vegetables), processed culinary ingredients (e.g., oils and salt), processed foods (e.g., canned vegetables and processed meats), and UPFs (e.g., packaged snacks, breakfast cereals, and baby formulas) (Table 1).

**Table 1:** NOVA classification system of foods Adapted from Monteiro CA, Cannon G, Lawrence M, Costa Louzada ML, Pereira Machado P. Ultra-Processed Foods, Diet Quality, and Health Using the NOVA Classification System. Food and Agriculture Organization of the United Nations; Rome, Italy: 2019 [11]. UPFs: ultra-processed foods

Group	Definition	Examples
Group 1: unprocessed or minimally processed foods	Unprocessed foods are edible parts of plants, animals, fungi, algae, or water, taken directly from nature. Minimally processed foods are natural foods that have been altered by simple methods, such as drying, boiling, or freezing, without the addition of any extra ingredients.	Fruits, vegetables, grains, legumes, roots, tubers, meat, seafood, eggs, milk, flours, nuts, seeds, herbs, spices, tea, coffee, water.
Group 2: processed culinary ingredients	These are substances derived from Group 1 foods or natural sources, processed through methods such as pressing or refining.	Oils, butter, sugar, molasses, honey, syrups, starches, salt, and products made from these (e.g., salted butter).
Group 3: processed foods	Made by adding salt, oil, or sugar to Group 1 foods, these products are processed to improve taste, texture, or shelf life.	Canned vegetables/legumes, salted or sugared nuts/seeds, processed meats/fish, canned fish, fruit in syrup, fresh breads and cheeses.
Group 4: UPFs	UPFs are industrial formulations created through multiple processing steps, often utilizing ingredients not commonly found in home kitchens.	Soft drinks, packaged snacks, sweets, ice cream, mass-produced breads, breakfast cereals, energy bars/drinks, flavored milk/yoghurts, instant sauces, ready meals, reconstituted meats, instant soups/noodles/desserts, and baby formulas.

Their affordability, convenience, and aggressive marketing have established them as dietary staples in high-income nations and are increasingly becoming so in middle- and low-income countries [12,13]. Growing evidence suggests that UPFs may contribute to cancer risk through several overlapping mechanisms [14,15]. These include the promotion of weight gain and obesity, exposure to carcinogenic additives and contaminants formed during processing, disruption of gut microbiota and endocrine signaling, and the induction of chronic systemic inflammation [14,16,17]. These factors may act in synergy or exert independent influences on carcinogenesis, highlighting the need to disentangle their specific contributions [14,18]. The diet-cancer relationship is inherently multifactorial, shaped by nutritional composition, overall dietary patterns, cultural determinants, and interindividual metabolic variability [4,19]. An expanding body of evidence implicates elevated UPF consumption in a wide range of adverse health outcomes, with consistent associations reported for increased risks of cancer, cardiometabolic disorders, and multimorbidity encompassing these conditions [20,21].

The pervasiveness and diversity of UPFs in modern diets make them particularly challenging to study, yet their potential impact on public health cannot be overstated. Beyond individual-level risk, the rise of UPFs is also a symptom of broader failures in the food system [8,9]. Current agricultural and food production models increasingly prioritize shelf-stable, ultra-processed commodities over fresh, whole foods, contributing to a global environment where healthy dietary choices are often inaccessible, unaffordable, or culturally deprioritized [8,9]. This context has major implications not only for cancer prevention but also for policy-making in areas such as food labeling, taxation, marketing regulation, and public health education [12]. This narrative review synthesizes evidence from cohort studies and systematic reviews published between 2010 and 2025, aiming to clarify the association between UPF consumption and cancer risk. It addresses gaps in previous reviews by integrating recent large-scale population data and evaluating differences across cancer types and processing categories.

## Review

Global consumption patterns

UPFs currently contribute between 20% and 60% of daily energy intake across populations worldwide [7,10]. In high-income countries such as the United States, the United Kingdom, and Canada, they account for more than half of total caloric intake [7,9,22]. Vegetable consumption in the typical American diet is declining, with an increasing reliance on processed, plant-based foods [7,12]. In 2021, a median of 24% of adults reported consuming two or more servings of fruit daily, while 11% reported eating three or more servings of vegetables per day [3]. Meanwhile, consumption in middle- and low-income countries is increasing rapidly, driven by globalization, urbanization, and socioeconomic pressures [6,7,9].

A recent data analysis revealed that UPFs are sold at significantly higher rates in North America, Western Europe, and Australia compared to other parts of the world. However, sales are rapidly increasing in regions such as Latin America, Eastern Europe, North Africa, the Middle East, and Central and Eastern Asia, and this growth is expected to continue in the coming years [23]. The increase in UPF consumption is tied to several features of today’s lifestyles, including extended eating periods, more frequent snacking, dining out more regularly, and the convenience and affordability that UPFs offer [23]. Marketing strategies, the expansion of multinational food corporations, and shifts in cultural eating habits further reinforce dependence on these products [12,13]. Demographic factors also play a role: UPF intake is disproportionately higher among younger individuals, working women, urban dwellers, and low-income families, particularly where fresh foods are less accessible [7,12].

Pathophysiological mechanisms

The potential carcinogenicity of UPFs arises from their nutrient profile, chemical additives, processing byproducts, and their ability to promote chronic inflammation [14,18].

Nutritional Composition

A higher intake of UPFs has been linked to a noticeable increase in free sugars, as well as total and saturated fats, and sodium. At the same time, it’s associated with lower levels of essential nutrients, including fiber, protein, potassium, zinc, magnesium, and vitamins A, C, D, E, B12, and niacin [23]. This imbalance contributes to systemic inflammation, dyslipidemia, and endothelial dysfunction, thereby playing a central role in the development of cancer [13]. Moreover, excessive heat treatment and disruption of the food matrix during processing can negatively affect gut microbiota composition, further amplifying inflammatory pathways [13].

Chronic consumption of high amounts of sugar raises insulin levels and suppresses the production of insulin-like growth factor-binding proteins 1 and 2 (IGFBP-1 and IGFBP-2). This suppression increases the availability of free IGF-1, resulting in prolonged activation of IGF-1 signaling in various tissues. Sustained IGF-1 activity persistently stimulates the RAS/RAF/MEK/ERK pathway, leading to uncontrolled cell proliferation [24]. At the same time, it promotes cell survival by inhibiting apoptosis through the PI3K/AKT pathway. IGF-1 signaling also enhances the expression of vascular endothelial growth factor, encouraging angiogenesis, and activates matrix metalloproteinases, which break down extracellular matrix components. Together, these effects drive cancer progression by supporting tumor growth, blood vessel formation, and metastasis [24].

Regarding the fat composition of UPFs, their pro-inflammatory effects stem not only from the larger quantities typically consumed compared to other foods, but also from the poorer quality of these fats. Industrially produced trans fatty acids, in particular, have been strongly linked to chronic low-grade inflammation and are associated with elevated levels of biomarkers such as high-sensitivity C-reactive protein, IL-6, and TNF-α [25]. Meta-analyses of total trans-fat intake suggest a notable association with certain cancers. Higher consumption was linked to an increased risk of prostate cancer (OR 1.49; 95% CI, 1.13-1.95) and colorectal cancer (OR 1.26; 95% CI, 1.08-1.46). In contrast, no significant associations were observed for breast cancer (OR 1.12; 95% CI, 0.99-1.26), ovarian cancer (OR 1.10; 95% CI, 0.94-1.28), or non-Hodgkin lymphoma (OR 1.32; 95% CI, 0.99-1.76) [26]. UPFs are also characterized by a high salt content, which contributes substantially to overall sodium intake [25]. Evidence from several cross-sectional studies has linked elevated salt consumption with increased CRP levels in adults and older populations, though this association has not been consistently observed in adolescents [25]. These disturbances create a pro-oncogenic environment primed for malignant transformation.

Additives and Processing Byproducts

Non-nutritional components of UPFs also contribute significantly to low-grade inflammation, a recognized contributor to chronic disease. Additives such as sweeteners (e.g., acesulfame potassium, sucralose, aspartame) and emulsifiers, while used to enhance sensory properties and extend shelf life, have been shown to disrupt gut homeostasis and immune regulation [24,25]. Emulsifiers, widely used in UPFs to keep ingredients blended, are increasingly being studied for their potential role in cancer development. Research shows that these additives can disturb the gut microbiota, weakening the intestinal barrier and allowing harmful microbes to enter. This process raises levels of pro-inflammatory molecules such as flagellin and lipopolysaccharide. Over time, the resulting chronic, low-grade inflammation interferes with the body’s normal control of cell growth and death, creating conditions that may favor cancer formation [24].

UPF can contain a variety of harmful chemical substances that remain after production, including nitrates, pesticides, and dioxins [24,27,28]. Some dangerous compounds with cancer-causing or gene-altering effects can also form during storage or when high-protein foods are cooked at high temperatures. These include byproducts of fat and protein oxidation, polycyclic aromatic hydrocarbons, heterocyclic aromatic amines, and nitroso compounds, which can develop from nitrates added to meats [29,30]. Titanium dioxide can disrupt gene expression and promote oxidative stress in animal models [24,30].

**Figure 1:**
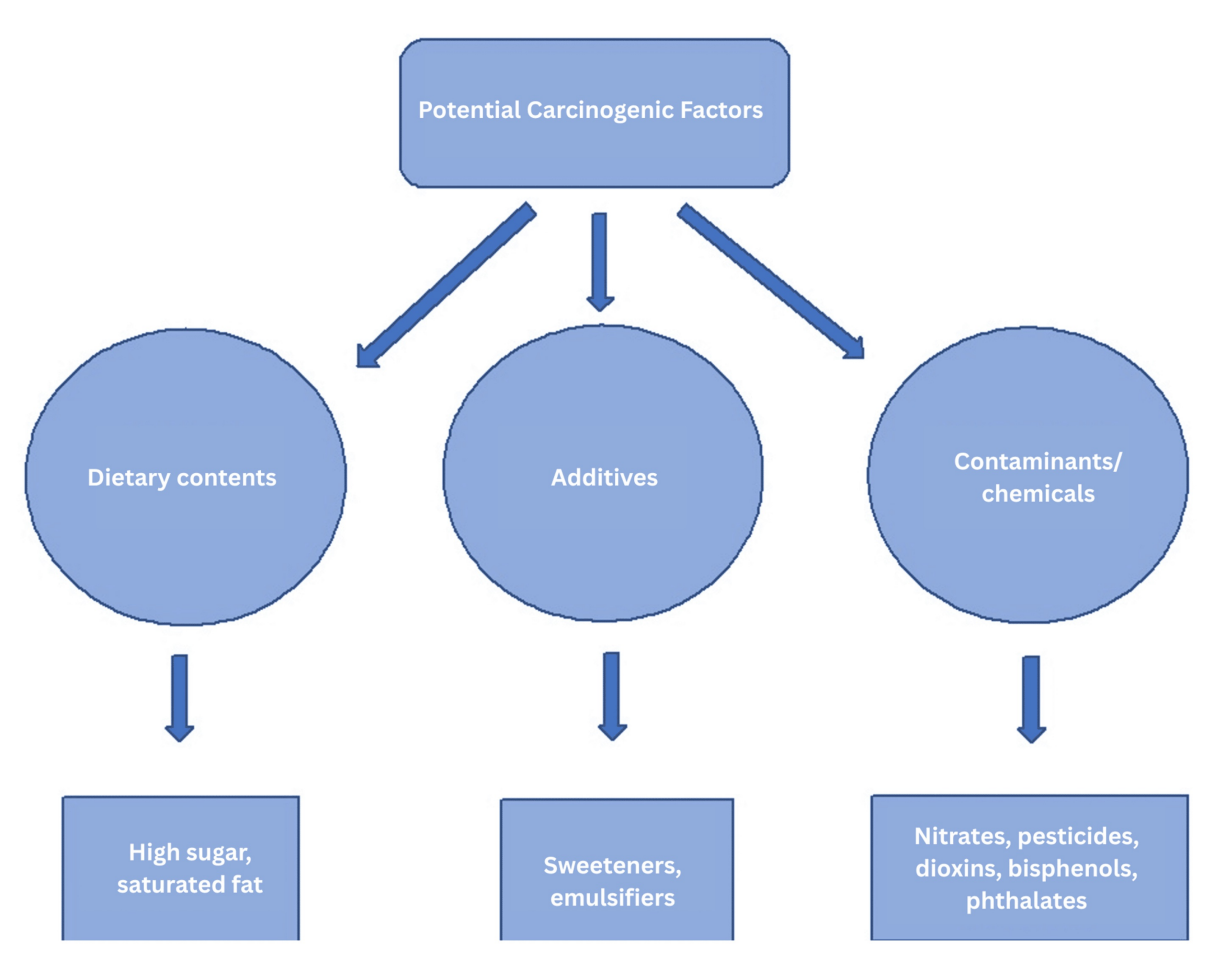
Potential carcinogenic factors underlying the association between UPFs and cancer risk UPFs: ultra-processed foods Image Credit: Author

Additionally, bisphenols and phthalates, chemicals that can migrate from food packaging into UPFs, are linked to endocrine disruption and inflammatory responses. These substances further contribute to oxidative stress, DNA damage, and dysregulated cell signaling, processes that underlie the development of cancer [24,25]. A cross-sectional study of the U.S. population in 2013-2014 found a stronger link between UPF consumption and higher levels of mono-(carboxyisoctyl) in the urine of children, a marker of exposure to diisononyl phthalate (DiNP). This is especially concerning because DiNP is known to have antiandrogenic effects, which can interfere with hormone function. DiNP is listed under California’s Proposition 65 as a chemical known to cause cancer, raising important health concerns, particularly for children [31,32]. The listing decision is largely supported by animal and mechanistic data (e.g., tumor formation in rodents, mechanistic pathways) rather than robust human epidemiologic studies [32]. In summary, high levels of sugar and saturated fats, as well as additives such as emulsifiers and sweeteners, and other chemicals, may help explain the observed association between UPF and cancer risk (Figure 1).

Microorganisms have shown great potential in helping to neutralize harmful mutagens by breaking them down through a process called biotransformation. As a result, there’s a growing need to identify and study microbes that can carry out this process [33]. To make food safer, various strategies have been developed to reduce the presence of harmful substances. These include pre- and post-processing treatments, changing ingredients (reformulation), adding protective additives, adjusting cooking conditions, and using advanced processing technologies. Together, these methods help prevent or minimize food-related hazards [34].

Chronic Inflammation and Microenvironmental Changes

UPFs contribute to chronic low-grade systemic inflammation, a key driver of carcinogenesis [13,14]. Chronic inflammation contributes to tumorigenesis by attracting inflammatory cells and releasing cytokines, chemokines, and proteases that promote DNA damage and genomic instability. These inflammatory mediators activate oncogenes, suppress tumor suppressor genes like p53, and interfere with normal cell cycle regulation [24]. Reactive oxygen species (ROS) and cytokines produced during inflammation further exacerbate DNA damage, triggering the abnormal expression of activation-induced cytidine deaminase and leading to mutations in key genes, such as TP53 and MYC. ROS also impair the function of checkpoint proteins ATM and ATR, compromising cell cycle arrest mechanisms. Additionally, inflammation suppresses the activity of mismatch repair proteins and disrupts the regulation of cyclins, thereby fostering genomic instability and unchecked cell proliferation. Collectively, these effects promote tumor progression and sustain a pro-inflammatory tumor microenvironment [24].

UPFs also promote dysbiosis, reducing the number of beneficial bacteria and favoring pathogenic species, which further drives inflammation [27,31]. These disruptions activate immune cells that secrete pro-inflammatory mediators, including TNF-α, IL-6, and IL-1β, and generate ROS that induce DNA damage and mutations [13,32]. Over time, this inflammatory and oxidative environment fosters a tumor-promoting microenvironment characterized by uncontrolled proliferation, angiogenesis, and immune evasion [13,15]. Collectively, these chemical, inflammatory, metabolic, and microbiome-mediated mechanisms converge to create a pro-oncogenic internal environment [14,18]. Through DNA damage, altered gene regulation, immune modulation, and sustained proliferative signaling, high UPF consumption substantially increases the risk of cancer initiation and progression [10,15,35].

The cancer-protective benefits of a plant-based diet may be largely due to its anti-inflammatory and antioxidant properties [29]. Plant-based foods are rich in beneficial compounds, including antioxidants, phytoestrogens, and flavonoids, especially flavanones, that can help slow or prevent cancer development by reducing inflammation, neutralizing harmful free radicals, or blocking the formation of cancer-causing substances. These foods also contain other cancer-fighting nutrients, such as fiber, folate, vitamin C, vitamin A, and beta-carotene [29]. Studies consistently show that consuming more fruits and vegetables is associated with a lower risk of several types of cancer, including those of the esophagus, lungs, stomach, and colon [29]. This highlights the need for dietary interventions and public health strategies aimed at reducing UPF intake [7,10,36].

Discussion

Dietary Modernization and Emerging Health Risks

In recent decades, UPFs have quietly become a dominant part of daily diets, appearing in a wide range of products, including breakfast cereals, snack bars, frozen meals, and sugary beverages. Confectionery classified as “cookies, pastries, and sweet bread,” along with sugar-sweetened beverages, are among the most widely consumed categories of UPFs [23]. While these foods provide convenience, a long shelf life, and affordability, mounting scientific evidence warns that they carry a hidden cost: a heightened risk of cancer. Large-scale prospective cohort studies provide compelling evidence of this risk. Kliemann et al. and Cordova et al. corroborate these findings, showing that high UPF intake is consistently associated with a higher cancer risk across multiple European populations, underscoring the global relevance of this issue [5, 37]. Cordova et al. tracked 266,666 initially disease-free participants across seven European countries over a median of 11.2 years. The researchers found that a 1-standard-deviation rise (approximately 260 g/day) in UPF intake was associated with a 9% increased risk of cancer-cardiometabolic multimorbidity [37].

In Kliemann et al., dietary intake was classified using validated food frequency questionnaires, and cancer incidence at 25 anatomical sites was tracked through national cancer registries and mortality databases, according to the NOVA system. The researchers applied Cox proportional hazards models to assess the impact of substituting 10% of daily intake from processed (NOVA 3) or UPFs (NOVA 4) with unprocessed or minimally processed foods (NOVA 1). The results showed that replacing 10% of processed food with minimally processed food was associated with a reduced risk of overall cancer. Strengths of the study include its large, multi-country sample, long follow-up, and robust adjustment for confounders. Limitations include potential measurement error from self-reported diets and residual confounding [5]. Drawing on data from international cohorts, global dietary surveys, and mechanistic studies, the authors in Kliemann et al. highlight the rapid worldwide expansion of UPF consumption and its links to obesity, metabolic dysregulation, and chronic inflammation, which are key risk factors for cancer [4]. These results provide strong epidemiological evidence supporting the role of UPFs in cancer development, underscoring the importance of dietary recommendations that prioritize minimally processed foods.

Nonetheless, the evidence remains inconsistent across different populations. For instance, Kityo and Lee, in a large Korean cohort study, found no significant overall association between total UPF intake and cancer mortality; however, the results may be influenced by reverse causation, such as participants reducing UPF intake after early symptoms of disease or residual confounding from unmeasured lifestyle or dietary factors. Additionally, certain subcategories, such as processed meats and flavored milk beverages, were linked to an increased risk. These inconsistencies may be explained by differences in cultural dietary habits, variability in UPF classification criteria, or population-specific genetic and environmental factors that influence susceptibility to dietary exposures. Crucially, this heterogeneity highlights the importance of developing context-sensitive dietary guidelines rather than relying on generalized risk assessments [38].

Still, questions remain. Future research must tease apart the specific contributions of food additives, high-temperature processing byproducts, and overall dietary patterns, while also considering genetic differences, lifestyle habits, and the role of the gut microbiome in shaping cancer risk. Such research will enable more refined recommendations that respect cultural food traditions and individual dietary preferences. A notable challenge in studies of UPF consumption is the potential for misclassification, often arising from incomplete information regarding the specific foods reported by participants. Nevertheless, the current body of evidence consistently indicates an association between higher consumption of UPF and increased cancer risk (Table 2).

**Table 2:** Key characteristics of reviewed studies UPFs: ultra-processed foods, CVDs: cardiovascular diseases, EPIC: European Prospective Investigation into Cancer and Nutrition, ER: estrogen receptor

Author	Year of publication	Type of study	Purpose of study	Results
Chang et al. [19]	2023	Prospective cohort	Investigates the relationship between UPF consumption and the risk and mortality of 34 different site-specific cancers.	Each 10% ↑ in UPF intake → +2% overall cancer risk, +19% ovarian cancer risk. Higher UPF intake linked to ↑ incidence of overall and brain cancer, ↓ head and neck cancer.
Babalola et al. [24]	2025	Literature Review	Examines available epidemiological, mechanistic, and clinical data on the association between UPF intake, CVDs, and cancer.	UPFs present substantial risks for cardiovascular disease and cancer owing to their poor nutrient profile and adverse metabolic effects.
Contaldo et al. [7]	2020	Literature Review	Examines food, health, and lifestyle from an ecological perspective, this review considers climate, demographics, the food system, and epigenetics to clarify the role of nutrition in cancer risk.	The association between cancer and climate change can be traced to the unsustainable food system established in recent decades.
Cordova et al. [37]	2023	Prospective cohort	Assess the relationship between overall and subgroup intake of UPFs and the prevalence of multimorbidity.	Higher UPF consumption associated with multimorbidity (cancer + CVD + diabetes). Stronger associations for sugary/ artificially sweetened beverages and animal-based UPFs.
Fiolet et al. [12]	2018	Prospective cohort	Evaluate the prospective relationship between UPF consumption and the risk of developing cancer.	10% ↑ in UPF intake → 12% higher overall cancer risk, 11% higher breast cancer risk.
Kityo and Lee [38]	2023	Prospective cohort	Investigated the relationship between UPF intake and mortality from all causes, cancer, and CVD.	UPF not significantly associated with all-cause, cancer-specific, or cardiovascular mortality. But ↑ UPF red meat, fish, flavored milk/soymilk linked to higher mortality (esp. men).
Kliemann et al. [4]	2022	Literature Review	Summarize and critically evaluate published research on the association between UPF consumption and cancer risk, including biological mechanisms.	Although limited, current epidemiological evidence suggests a positive association between the consumption of UPFs and the risk of certain types of cancer.
Kliemann et al. [5]	2023	Prospective cohort	The association between food intake, classified by processing level, and cancer risk at 25 anatomical sites was investigated using data from the EPIC study.	Replacing 10% of UPFs with minimally processed foods reduced the risk of multiple cancers (colon, rectal, head/neck, breast, etc.).
Lian et al. [14]	2023	Systematic review	Provides a systematic review examining the links between UPF intake and various types of cancer.	Each 10% ↑ in UPFs → 4% higher colorectal cancer risk (esp. men), higher breast cancer risk (varied by menopausal status and ER subtype).
Menegassi and Vinciguerra [15]	2025	Literature Review	Assesses the role of UPFs in cancer risk and improved preventive approaches.	Findings indicate strong links between high UPF intake and increased risk of colorectal, breast, and overall cancer.
Yang [13]	2025	Literature Review	Aims to summarize the associations between different foods and cancer.	Cancer and diet are closely linked, warranting further study of underlying mechanisms and strategies for improved prevention and treatment.

How UPF Influences Cancer Development

Understanding how UPFs contribute to cancer requires examining the underlying biological mechanisms that drive this process. Menegassi and Vinciguerra highlighted that UPFs may promote carcinogenesis through chronic inflammation, oxidative stress, gut microbiota dysbiosis, and metabolic dysregulation, as well as exposure to additives and processing-related contaminants. The review gave particular attention to the role of chemical additives (such as emulsifiers, artificial sweeteners, titanium dioxide, nitrites, and acrylamide) and high-temperature processing byproducts (heterocyclic amines and polycyclic aromatic hydrocarbons) in generating genotoxic and mutagenic effects. Mechanisms such as DNA adduct formation, epigenetic alterations, impaired apoptosis, and activation of pro-oncogenic pathways (PI3K/AKT/mTOR, MAPK) were highlighted as critical contributors to tumor initiation and progression. It also discussed how these mechanisms intersect with obesity and other metabolic disorders, which are established cancer risk factors [15].

Drawing on epidemiological data, mechanistic research, and clinical findings, Babalola et al. demonstrate that UPFs, characterized by high levels of unhealthy fats, refined sugars, sodium, and synthetic additives, contribute to major risk factors such as obesity, hypertension, and dyslipidemia, thereby increasing the risk of cardiovascular disease and cancer. This study emphasizes that, mechanistically, chronic UPF intake drives systemic inflammation, oxidative stress, and endothelial dysfunction, creating a biological environment conducive to disease progression. Certain additives, including artificial sweeteners and sodium nitrites, are further implicated in the induction of genotoxicity and pro-inflammatory microenvironments that may facilitate carcinogenesis [24].

Lian et al. highlighted several plausible biological mechanisms underlying these associations. The consumption of UPFs may elevate exposure to endocrine-disrupting chemicals such as bisphenol A and phthalates, which can induce persistent epigenetic modifications and stimulate the proliferation of hormone-sensitive tissues in a tumorigenic manner. Additionally, UPFs may unfavorably alter the composition and function of the gut microbiota, thereby increasing cancer risk through various molecular pathways, including the suppression of T-cell activity and the promotion of DNA damage. Strengths of this work include comprehensive literature coverage and quantitative synthesis. However, limitations include heterogeneity across studies and reliance on self-reported dietary data [14].

Yang integrates evidence from nutritional epidemiology with emerging data from molecular and cellular studies to highlight how dietary choices interact with genetic susceptibility and lifestyle factors. The review highlights the significance of diet quality throughout the cancer continuum, from prevention to survivorship. It emphasizes the impact of dietary patterns on shaping systemic metabolic and inflammatory states that either promote or protect against carcinogenesis [13]. Contaldo et al. also highlighted epidemiological evidence showing correlations between Westernized diets and increased incidence of cancers such as breast and gastrointestinal malignancies. By combining mechanistic insights with population-level data, the review highlights the importance of promoting traditional, nutrient-rich dietary patterns and implementing public health strategies to mitigate the growing global burden of diet-related cancers [7].

Site-Specific Evidence of UPF-Related Cancer Risk

The relationship between UPFs and cancer is increasingly recognized as both significant and site-specific. Breast cancer is the most common cancer among women in the United States and is strongly influenced by environmental factors, particularly diet [3,13]. Yang underscores that research shows that certain foods and nutrients can either help protect against or contribute to breast cancer by altering the body's epigenetic makeup, affecting gene activity, non-coding RNA, histone modifications, and even gut microbiome metabolism [13]. Chang et al., analyzing data from nearly 200,000 adults in the UK Biobank over almost a decade, found that each 10% increase in UPF intake was associated with a 2% increase in overall cancer risk, with particularly significant rises in breast and ovarian cancer incidence and mortality. This study’s strength lies in its long follow-up, large sample size, and detailed cancer-specific analyses, though its observational design limits causal inference and may be affected by dietary misreporting and population characteristics [19]. Similarly, Fiolet et al. investigated the association between the consumption of UPF and cancer risk in the NutriNet-Santé prospective cohort, comprising 104,980 French adults (median age, 42.8 years) over a median follow-up period of five years. There were 2,228 incident cancer cases documented, including 739 cases of breast cancer. Participants were stratified into sex-specific quartiles of UPF consumption, and their baseline characteristics, including age, education, smoking status, physical activity, and energy intake, were compared. The analysis revealed that a 10% increase in UPF consumption was associated with a 12% higher risk of overall cancer and an 11% increased risk of breast cancer [12].

Evidence from a study by Yang demonstrates that higher consumption of UPFs, characterized by elevated fat content and reduced dietary fiber, correlates with increased breast cancer incidence. The underlying mechanisms likely involve adiposity-driven estrogen excess, systemic inflammation, and metabolic dysregulation. In contrast, dietary patterns emphasizing traditional and fermented foods appear protective, with potential benefits mediated through improved microbiota composition, reduced inflammatory signaling, and enhanced metabolic stability [13].

Colorectal and other digestive system cancers also exhibit strong and consistent associations with UPF intake. Several biological mechanisms may help explain these associations, including the poor nutritional quality of UPFs, the inclusion of harmful additives, the formation of contaminants during processing, and the disruption of gut microbiota balance [15]. Meanwhile, Lian et al. emphasize the role of high sugar, refined starch, and saturated fat content in altering gut microbiota, impairing epithelial integrity, and promoting a pro-inflammatory microenvironment [14]. Taken together, these processes establish a biologically plausible link between UPFs and gastrointestinal carcinogenesis. While some studies suggest that organic food consumption may be associated with a lower risk of cancers, such as stomach cancer, overall findings remain limited and mixed [13]. In cancers such as lung and gastrointestinal types, poor appetite and low intake of protein and energy are common and contribute to weight loss and cachexia, highlighting the need for early nutritional support. Chronic inflammation, which can be influenced by diet, is a key driver of primary liver cancer; diets rich in anti-inflammatory foods may help reduce risk and mortality [13].

Overall, current evidence points to a strong, though complex, relationship between UPFs and cancer risk, particularly for breast and colorectal cancers. While some studies report null findings, reflecting the challenges of disentangling dietary exposures from cultural and biological factors, the recurring associations across varied populations underscore the potential benefits of reducing UPF consumption. Looking ahead, adopting more consistent methods for classifying UPFs and conducting research that accounts for cultural and genetic diversity will be crucial for refining dietary guidelines and translating epidemiological insights into practical public health strategies.

Worldwide Trends and Implications of UPF Intake

The rise of UPFs is not just a dietary shift but part of a much larger story of how globalization and modernization are reshaping what people eat. Contaldo et al. remind us that in many low- and middle-income countries, rapid urbanization and increasing exposure to global food markets have led communities away from traditional diets centered on grains, legumes, and fresh produce. Instead, families are increasingly turning to Western-style convenience foods, packaged snacks, sugary drinks, and processed meats, which are affordable and easily accessible but often nutritionally deficient. The result has been a visible surge in obesity, diabetes, and diet-related cancers, even in regions where undernutrition and food insecurity persist. This double burden highlights the unique vulnerability of populations caught in the midst of the nutrition transition [7].

Addressing these challenges requires more than simply urging individuals to “eat better.” As Babalola et al. argue, education campaigns are essential for raising awareness of the risks of UPFs, but they cannot succeed in isolation. People make food choices within environments shaped by price, availability, and marketing [24]. Yang echoes this point, emphasizing that structural measures, such as food labeling, regulating additives, restricting aggressive advertising, and promoting minimally processed foods, are crucial to creating healthier food systems. Without these changes, the cheapest and most visible options will remain the very products that increase cancer risk and other chronic illnesses [13,24].

At the same time, it is essential to acknowledge the cultural significance of food. Traditional dietary practices and local foodways are not only healthier but also deeply tied to identity and community [13]. Protecting these practices through supportive policies and education initiatives can provide both a nutritional and cultural safeguard against the rising prevalence of UPFs [13,24]. Taken together, these studies suggest that the path forward must blend awareness with accessibility: empowering individuals to make healthier choices while ensuring that healthier foods are also the easiest, most affordable, and most appealing options available.

Clinical and Public Health Relevance With Future Research

Several countries, including Brazil, Canada, and France, now recommend limiting UPFs in national dietary guidelines. Global health bodies, such as the Food and Agriculture Organization and the World Cancer Research Fund, concur with this, recommending that people replace UPFs with whole foods, including fruits, vegetables, whole grains, and legumes, to reduce the risk of chronic diseases [4]. While cutting back on UPFs is key, improving how they're made could mitigate some of the harm. New technologies may help create healthier, eco-friendly options, but these changes require strong government oversight; voluntary industry efforts have proven ineffective. Stronger public policies are also needed, such as limiting UPF marketing and supporting local fresh food producers, to make healthy diets more accessible and reduce both health and environmental damage from UPFs [4].

UPF industry groups have worked to influence the World Health Organization (WHO) policies on chronic diseases by forming alliances, gaining insider access, and promoting industry-friendly messages. These tactics are similar to those once used by the tobacco industry to block public health action. To protect global efforts for healthier diets, stronger rules are needed to prevent industry interference, both at the WHO level and within national governments [35]. On an individual level, the research also offers hopeful pathways. Studies highlight that reducing UPF intake and replacing it with whole, nutrient-rich foods, such as fruits, vegetables, legumes, whole grains, and traditional fermented foods, can protect against cancer while also improving metabolic and immune health. These findings remind us that prevention is not only about avoiding harm but also about restoring balance through dietary practices that nourish both body and culture [13,15].

The combined insights from mechanistic, epidemiological, and nutritional research make one message clear: action on UPFs can no longer be delayed [27,28,29]. Laboratory studies reveal how additives, contaminants from processing, and nutrient imbalances disrupt biological systems, thereby fostering cancer development [24]. At the same time, large population studies consistently show that people who eat more UPFs face higher risks of cancer and other chronic diseases [5,12,19,37]. Taken together, this evidence presents a compelling case for the implementation of urgent public health measures. Policies such as front-of-package labeling, restrictions on food advertising (particularly to children), taxation of unhealthy products, and investment in local food systems that prioritize fresh, minimally processed foods have all shown promise [4,13]. Chile’s mandatory warning labels on high-sugar, high-fat, and high-sodium foods, combined with the United Kingdom's tiered tax on sugar-sweetened beverages, demonstrate how governments can effectively combine regulatory and fiscal tools to curb the consumption of UPFs and improve population health outcomes [39,40]. Importantly, these strategies acknowledge that food choices are influenced by factors such as affordability, accessibility, and marketing contexts [7,13]. By modifying these environmental determinants, the adoption of healthier dietary patterns can be facilitated in a more equitable and achievable manner [7].

Limitations

As a narrative review, this work did not adhere to a predefined set of inclusion or exclusion criteria as in systematic reviews, which inherently limits reproducibility and transparency. Only studies published in English were considered, introducing a potential selection bias, as relevant findings published in other languages may have been excluded. This language restriction could skew the overall conclusions in favor of English-speaking populations or research contexts. Additionally, the review may be subject to publication bias, since studies reporting significant or positive associations between UPF intake and cancer risk are more likely to be published than those with null results.

The absence of a structured review protocol also limits reproducibility, as another researcher might not retrieve or interpret the same body of evidence using identical methods. Moreover, heterogeneity in exposure definitions was noted among included studies, particularly regarding the application of the NOVA classification system. Some studies employed modified NOVA criteria, while others classified foods differently based on available dietary data, which complicates direct comparisons and the synthesis of results. Given these limitations, future work employing systematic review or meta-analytic methods could complement these findings and help provide a more rigorous, reproducible, and quantitative assessment of the relationship between UPF consumption and cancer risk.

## Conclusions

UPFs, often high in additives and low in nutritional value, have become a staple in many people’s daily diets. While convenient and widely available, these foods are playing a significant role in the rise of non-communicable diseases around the world, including cancer. A growing number of studies have linked high consumption of UPFs to increased risks of cancers such as colorectal and breast cancer. This may be due to a combination of factors, including weight gain, exposure to harmful additives and substances created during processing, changes in gut health, and chronic inflammation in the body.

Cutting back on UPFs and choosing minimally processed, whole-food alternatives could be a powerful step toward reducing cancer risk. However, making that shift on a large scale requires more than individual willpower; it calls for strong public health policies. These might include clearer food labeling, taxes on unhealthy products, stricter marketing regulations (especially for children), and broader reforms in agriculture and early-life nutrition. At the same time, more in-depth research is needed to understand exactly how these foods affect the body at a molecular level and to clarify their role in cancer development. Ultimately, addressing the health risks associated with UPFs will require a collective effort, combining scientific research, effective policies, and public awareness, to create food environments that support healthier choices and help reduce the global burden of cancer.
